# Genome assembly of *Thaumatotibia leucotreta*, a major polyphagous pest of agriculture in sub-Saharan Africa

**DOI:** 10.1093/g3journal/jkac328

**Published:** 2022-12-13

**Authors:** Anandi Bierman, Minette Karsten, John S Terblanche

**Affiliations:** Centre for Invasion Biology, Department of Conservation Ecology & Entomology, Stellenbosch University, Stellenbosch 7600, South Africa; Department of Conservation Ecology & Entomology, Stellenbosch University, Stellenbosch 7600, South Africa; Centre for Invasion Biology, Department of Conservation Ecology & Entomology, Stellenbosch University, Stellenbosch 7600, South Africa

**Keywords:** *Thaumatotibia leucotreta*, hybrid genome assembly, agricultural pest, polyphagous

## Abstract

The false codling moth (FCM; *Thaumatotibia leucotreta*, Meyrick; Lepidoptera: Tortricidae) is a highly polyphagous, major agricultural pest indigenous to sub-Saharan Africa. With growing international trade, there is an increasing concern about introducing this pest into other countries. In South Africa, FCM poses a risk to multiple crops, and is currently suppressed through a combination of chemical, microbial, cultural, augmentative biological control, and the sterile insect technique. Compared with other lepidopteran agricultural pests, such as codling moth *Cydia pomonella*, genetic and other -omic resources for FCM have not been as well developed and/or not made publicly available to date. The need to develop genomic resources to address questions around insecticide resistance, chemosensory capabilities, and ultimately, develop novel control methods (e.g. gene editing) of this pest is highlighted. In this study, an adult male was sequenced using long-read PacBio Sequel II reads and Illumina NextSeq short reads and assembled using a hybrid assembly pipeline and Pilon error correction. Using the chromosome-level genome assembly of *Cy. pomonella*, we performed comparative analysis, arranged FCM scaffolds to chromosomes, and investigated genetic variation related to insecticide resistance and chemosensory capabilities. This work provides a platform upon which to build future genomic research on this economically important agricultural pest.

## Introduction

**Fig. 1.**
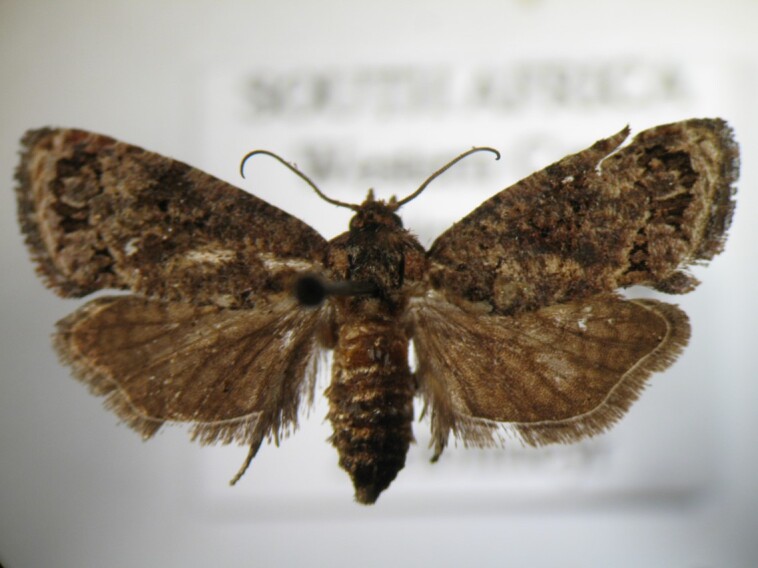
The adult false codling moth, *Thaumatotibia leucotreta* is an important polyphagous pest native to sub-Saharan Africa. Picture credit: P. Addison and C. Kapp.

The false codling moth (FCM), *Thaumatotibia leucotreta* (Meyrick) (Lepidoptera: Tortricidae) (Figure 1) is an important polyphagous pest native to sub-Saharan Africa (Prinsloo and Uys, 2015). It is a major concern for crop production in South Africa. Due to previous interceptions (Gilligan *et al*. 2011; Mazza *et al*. 2014), it is considered as a phytosanitary pest for all exports from South Africa, with potentially significant economic impacts especially on market access. This economic impact has led to many studies investigating the biology, ecology, and physiology of the species with applied management foci (e.g. Karsten *et al*. 2019; Huisamen *et al*. 2022).

An array of control options are employed for FCM management, such as mating disruption, the sterile insect technique (SIT), insecticides, and granulosis virus cover sprays (Moore and Hattingh 2012; Hofmeyr *et al*. 2015), usually implemented in combination as part of an Integrated Pest Management (IPM) program (Moore 2021). A key mechanism for population control is monitoring of the population through baited traps with pheromone-based lures (Adom *et al*. 2021). The chemosensory system in insects dictates behaviors such as location of food and mates or oviposition sites and, with application to IPM, the attractiveness to synthetic lures. Understanding the chemosensory capabilities of FCM can unlock novel approaches for population control through the development of chemical lures or interference with olfaction. Little is known of the chemosensory capabilities of FCM. However, it has been studied at the genetic level in other lepidopteran pests such as *Carposina sasakii*, the peach fruit moth (Tian *et al*. 2018) and *Chilo crambidae*, a lepidopteran pest of sugarcane (Liu *et al*. 2021) making the molecular mechanisms of odor reception accessible. Being able to attract specific sexes and understanding the basis of sex pheromones and kin recognition are key biological processes influencing population dynamics and highly relevant to IPM strategies for population control such as SIT (Hofmeyer *et al*. 2015) and mating disruption using semiochemicals (El-Ghany 2019).

The large-scale application of insecticides has resulted in reports of the development of insecticide resistance in FCM (Hofmeyr and Pringle 1998), which has been demonstrated in many other lepidopteran species, including *Plutella xylostella* (L.) (Lepidoptera: Plutellidae), *Spodoptera frugiperda* (Lepidoptera: Noctuidae), and *Cydia pomonella* (Lepidoptera: Tortricidae; Reyes *et al*. 2007; Neto *et al*. 2016; Lira *et al*. 2020). In the study by Wan *et al*. (2019), genetic variants identified in the genome of the closely related tortricid, *Cy. pomonella* (GCA_003425675.2) increased the susceptibility of moths to insecticides (deltamethrin and azinphos methyl) when silenced in insecticide-resistant individuals. This highlights the potential of an assembled genome as a potentially valuable resource in IPM.

Here, we present the first genome of the FCM, *Th. leucotreta*, assembled through a hybrid assembly pipeline using Illumina NextSeq reads and PacBio Sequel II long reads. Through reference to the chromosome-level genome assembly of *Cy. pomonella*, we performed a comparative analysis, linking FCM scaffolds to *Cy. pomonella* coding sequences and also investigated genetic variation related to IR and chemosensory capabilities. The contiguous, high-quality genome presented here is an essential genetic resource for the IPM of FCM.

## Materials and methods

### Organism origin

Adult male FCM were obtained from the mass-reared colony maintained by XSIT [X Sterile Insect Technique (Pty) Ltd. (XSIT)]. XSIT mass rears FCM for SIT release and regularly supplements their population with wild individuals to maintain higher genetic diversity. Moth samples were sent to Inqaba BioTec (Gauteng, South Africa) for DNA extraction and sequencing.

### Sequencing methods and preparation

High Molecular Weight (HMW) gDNA was extracted using Circulomics NanoBind Tissue Big DNA kit (PacBio, CA, USA). gDNA was sheared to ∼10–15 kb fragments using Covaris g-Tube (Covaris, LLC). For long-read sequencing, the Pacbio SMRTbell Library was prepared according to the manufacturer's protocol using the SMRTbell express template prep kit 2.0 (PacBio). Quality control was performed using Qubit HS dsDNA assay kit and TapeStation for library integrity check. Sequencing primer annealing and Polymerase Binding were carried out using the Sequel II binding kit 2.0, Internal Control 1.0, and Sequencing Primer v4. The bound complex was sequenced on a Sequell II system running SMRTcell 8M. For short read sequencing, fragment lengths were verified using the Bioanalyzer (Agilent Technologies, Palo Alto, CA, USA). The sequencing library was prepared using a MisSeq library prep kit according to the manufacturer's instructions. Library fragments were sequenced on the Illumina NextSeq sequencing platform.

### Sequencing reads processing

Both PacBio long reads and Illumina NextSeq short reads were checked for quality using FASTQC v0.11.5 (http://www.bioinformatics.babraham.ac.uk/projects/fastqc/). Low-quality bases were trimmed from the Illumina reads using FASTX-Toolkit v0.0.14 (http://hannonlab.cshl.edu/fastx_toolkit/), while Illumina adapter sequences were trimmed from Illumina reads using Trimmomatic v0.36 (Bolger *et al*. 2014). PacBio long reads were left untrimmed. Prior to read assembly, k-mer counting was conducted using Kmergenie v1.7016 (Chikhi and Medvedev 2013). K-mer histograms were used as input to GenomeScope (Vurture *et al*. 2017) in order to estimate heterozygosity and genome size.

### Sequence assembly

For genome assembly, each assembly step was verified for quality using QUAST v5.0 (Gurevich *et al*. 2013). PacBio long reads were assembled using Canu v2.1.1 (Koren *et al*. 2017) followed by mapping of trimmed Illumina reads to the PacBio Canu assembly using Burrows–Wheeler alignment tool (BWA-mem v 0.7.13; Li and Durbin 2010). Unmapped Illumina reads were extracted using Samtools v1.10 (Li *et al*. 2009). Unmapped Illumina reads were then assembled using SPAdes v3.13.0 (Bankevich *et al*. 2012). The PacBio Canu assembly and unmapped Illumina reads assembly were concatenated and the final assembly was run through Pilon v1.23 (Li *et al*. 2009) for error correction. Purge Haplotigs (Roach *et al*. 2018) was used to compound syntenic pairs of contigs caused by heterozygosity in the genome and often resulting in high duplication levels and inflated genome size. Gene space completeness was verified using BUSCO v5.0 (Simão *et al*. 2015) against a set of conserved genes from lepidoptera and the graphical representation was drawn using R (v.4.1.2).

### Exploratory annotation

The annotation of the FCM assembly was performed using the Maker annotation pipeline (v2.31.10; Cantarel *et al*. 2008). No ab initio gene prediction was implemented but a supported gene prediction strategy was employed. To do so, protein homology evidence in the form of the peptide sequence file in fasta format of the *Cy. pomonella* genome assembly (codling moth; Wan *et al*. 2019; GCA_003425675.2) was provided as input to the Maker pipeline in addition to EST homology evidence in the form of the CDS sequences (in fasta format) from the *Cy. pomonella* genome assembly (codling moth; Wan *et al*. 2019; GCA_003425675.2). The FCM assembly was mapped to the chromosome-level *Cy. pomonella* assembly (GCA_003425675.2) using Burrows–Wheeler alignment tool (BWA-mem v 0.7.13) and mapping coverage was tabulated using bedtools v2.27.1 *genomecov.* Graphical representation of the genome coverage was constructed using the R *circlize* package (Gu *et al*. 2014). RepeatMasker (v 4.0.7; Hubley and Green 2013) was used to identify repeat content in the assembly using default settings.

### Odorant reception and insecticide resistance

To investigate agriculturally significant traits in the FCM assembly, BLASTn and BLASTp (v2.4.0+; Altschul *et al*. 1990) were used against the list of coding sequences (CDS) from *Cy. pomonella* (codling moth; GCA_003425675.2; Wan *et al*. 2019). Specific focus was given to odorant receptor (OR) genes and Insecticide Resistance (IR) genes identified in *Cy. pomonella*. BLAST hits were limited to lepidopteran sequences through the use of the *-gilist* function and the lepidoptera.gi database. BLAST hits were filtered for sequence identity (>75%), *e*-value and sequence length and gene IDs were obtained through Entrez batch search (https://www.ncbi.nlm.nih.gov/sites/batchentrez) for highly similar (>99% sequence identity) BLAST hits.

## Results and discussion

Forward and reverse Illumina NextSeq reads had a length of 151 bp per read and 110,554,613 reads were obtained after sequencing with a GC content of 37%. PacBio Sequel II read length ranged up to 29,294 bp and 1,607,903 reads were obtained with a GC content of 37%. After trimming of low-quality bases from Illumina NextSeq reads, read lengths ranged from 50 to 104 bp for forward reads and 50 to 89 bp for reverse reads. A total of 110,010,916 reads remained for both forward and reverse sequences after trimming. Sequencing statistics for the raw Illumina and PacBio reads are summarized in Table 1 and compared with reads of *Cy. pomonella* (Wan *et al*. 2019) and *Bombyx mori* (Kawamoto *et al*. 2019). For the PacBio reads in particular, the FCM sequencing data set has only a fifth of the total number of reads compared with the *Cy. pomonella* and *B. mori* data sets. This provides some explanation as to the low coverage and difficulty in obtaining heterozygosity estimates as discussed below.

**Table 1.** Sequencing statistics for *Th. leucotreta*, *Cy. pomonella* (GCA_003425675.2; Wan *et al*. 2019), and *Bombyx mori* (GCF_014905235.1; Kawamoto *et al*. 2019).

Sequencing statistics	*Th. leucotreta*	*Cy. pomonella*	*B. mori*
# Raw Illumina reads	110,554,613	299,468,924,456	186,007,838
#Raw PacBio Reads	1,607,903	5,422,850	5,422,487

Supplementary Figure 1 depicts the k-mer profile of best K of 19 for Illumina short reads. However, as the graphic is not concave with a clear global maximum, the predicted best K and therefore, estimated genome size and heterozygosity could not be determined as the models using GenomeScope did not converge. Therefore, estimated genome size was based on the assembly size of 804 Mb (Table 2) and sequencing coverage was calculated at 10× coverage (Supplementary Table 1).

**Table 2.** Assembly statistics derived through quast for the false codling moth, *Th. leucotreta*, *Cy. pomonella* (GCA_003425675.2; Wan *et al*. 2019), *Tr. ni* (GCF_003590095.1; Fu *et al*. 2018), and *B. mori* (GCF_014905235.1; Kawamoto *et al*. 2019).

Genome statistics	*Th. leucotreta*	*Cy. pomonella*	*Tr. ni*	*B. mori*
Assembly size (bp)	804,903,742	772,891,954	368,200,000	460,334,017
Scaffold (>500 bp)*^a^*	14,831	1,717	1,031	696
Largest scaffold (bp)	1,806,666	34,601,981	NA	21,465,692
N50 (bp)	74,223	8,915,549	14,200,000	16,800,000
BUSCO genes (%)	93.1	97.8	97.5	95.5
Repeat (%)	12.88	42.87	20.5	43.6
G + C (%)	37.61	37.43	35.6	37.7
Number of genes	14,395*^b^*	17,184	14,043	14,623

N's per 100 kbp for the assembly was 0.00.

Number of unique *Cy. pomonella* peptide sequences matched to FCM assembly scaffolds in Maker output.

**Fig. 2.**
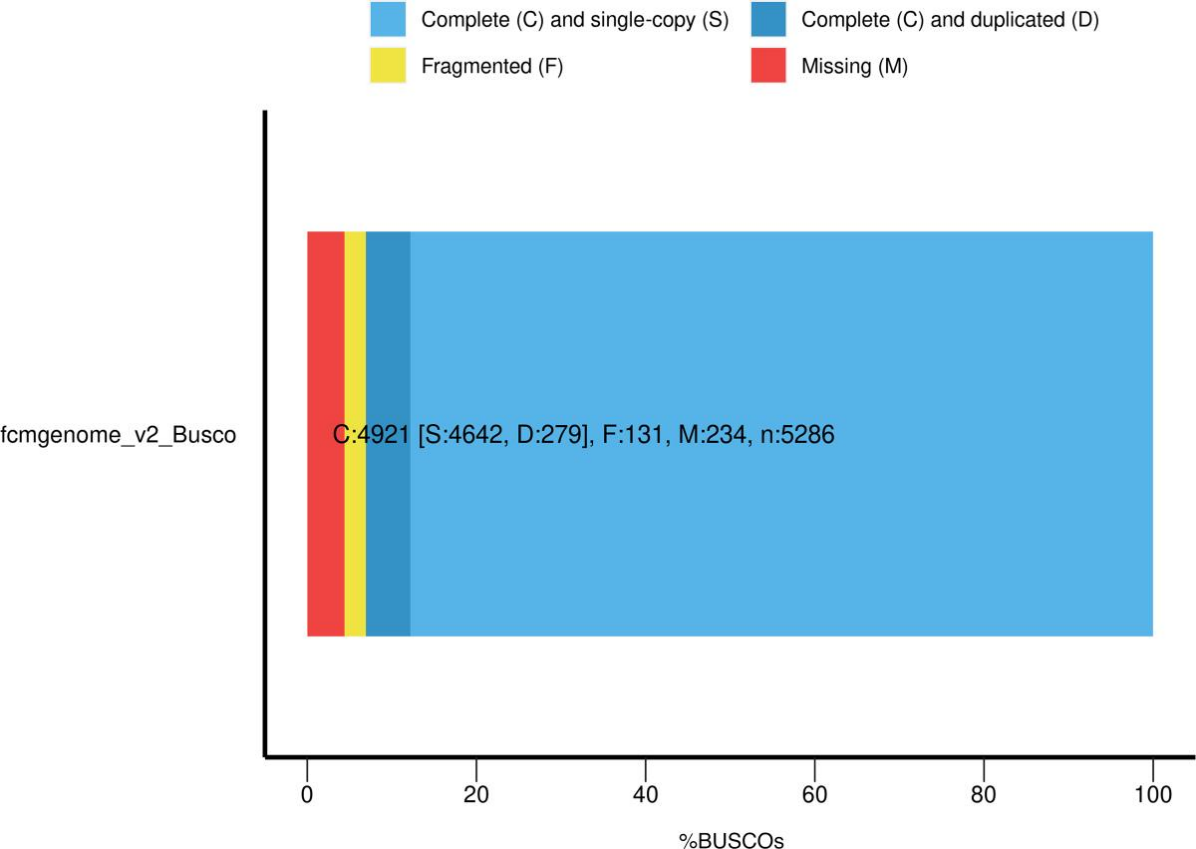
BUSCO assessment of assembly completeness for false codling moth, *Thaumatotibia leucotreta*, where: C = complete (93.1%), S = complete and single copy (87.8%), D = complete and duplicate (5.3%), F = fragmented (2.5%), and M = missing (4.4%).

Assembly statistics for the final Pilon corrected and purged assembly are summarized in Table 2 and compared with genomes of *Cy. pomonella*, *B. mori*, and *Trichoplusia ni*. Gene space completeness assessed through BUSCO yielded 93.1% complete BUSCOs of which 87.8% were complete and single copy, whereas 5.3% were complete and duplicated. Fragmented BUSCOs made up 2.5%, whereas missing BUSCOs made up 4.4% (Figure 2). Compared with the assembly size of *Cy. pomonella*, the current FCM assembly size is comparable as is the GC content. Similar to the related genomes depicted in Table 2, the BUSCO value for the FCM assembly is >90%. The low N50 value of the FCM assembly compared with related genomes could be attributed to the low-sequencing coverage (10×) of the FCM data set (Table 1).

In total, 58,214 repeat sequences were identified spanning 165,292,025 bp and constituting 12.88% of the assembly (Table 2). The majority of repeats identified consisted of LINEs (7.02%; Supplementary Table 2).

Genome coverage of the chromosome-level assembly of *Cy. pomonella* (GCA_003425675.2) showed uniform coverage of the entire genome by the FCM assembly with intermittent higher levels of coverage likely represented by low complexity or repetitive gene regions (Supplementary Figure 2).

The Maker annotation pipeline using protein and EST homology-based evidence from the *Cy. pomonella* genome assembly (GCA_003425675.2; 17,184 CDS sequences from the genome of *Cy. pomonella)* yielded matches to *Cy. pomonella* peptide sequences for 12,865 unique FCM scaffolds and matched 14,395 unique *Cy. pomonella* peptide sequences (Table 2; Supplementary Table 3). A total of 1,413 5′ UTRs and 2,049 3′ UTRs were identified matching 789 and 1,012 unique FCM scaffolds, respectively.

Of the 44 *Cy. pomonella* ORs available on GenBank, 23 had BLAST hits to the FCM assembly. Percentage identity ranged from 73 to 100% and *e*-values ranged from 0.0 to 9.92e-09. Though CpomOR3 was not identified in the FCM assembly, the co-receptor Orco was found. CpomOR3 is responsible for the allure to pear ester in *Cy. pomonella*. This might imply that pear ester is not attractive for FCM and that the Orco co-receptor interacts with some other OR gene in the absence of OR3 in FCM.

Several insecticide resistance-related genes have been identified in *Cy. pomonella* and SNPs identified between insecticide-resistant and susceptible strains of *Cy. pomonella* have been characterized (Wan *et al*. 2019). In the current assembly, one such SNP (G207C) in the Octopamine receptor gene was identified in a matching FCM scaffold. Table 3 lists the BLAST hit results to the *Cy. pomonella* OAR1 CDS (CPOM08177) and the associated SNP.

**Table 3.** BLAST hit results of *Cy. pomonella* OAR1 CDS to scaffold of *Th. leucotreta* (FCM scaffold) and the associated SNP.

Gene ID	Gene annotated	FCM scaffold	*e*-value	Percentage identity	Chromosome	Site	Nucleotide variation	Amino acid variation	FCM variant
CPOM08177	Octopamine receptor (OAR1)	tig00000302_pilon	0.0	83.22	21	7,548,673	G207C	V73L	G207C on scaffold tig00000302_pilon

The polyphagous nature and emerging insecticide resistance of FCM have made it a challenging pest causing devastation to South African agriculture and threatening the export market that drives a prominent part of this sector. Research focused on IPM strategies tailored to this pest and aimed at slowing the rise of insect resistance are prominent at the moment and there is a definite need for a quality genome assembly to aid in understanding the underlying biology of this lepidopteran pest. Studies currently under way include whole-transcriptome sequencing to improve the annotation of the genome with a specific focus on insecticide resistance as well as whole-genome SNP analyses to understand population genetics and potential host races.

## Supplementary Material

jkac328_Supplementary_Data

## Data Availability

This Whole-Genome Shotgun project has been deposited at DDBJ/ENA/GenBank under the accession JALPQI000000000. The version described in this paper is version JALPQI010000000. All scripts and associated parameters are available on GitHub (https://github.com/abtsubaki/FCMassembly). Supplemental material available at G3 online.
