# Supplementary material for: Genome assembly of Thaumatotibia leucotreta, a major polyphagous pest of agriculture in sub-Saharan Africa
Source: G3 (Bethesda). 2022 Dec 13;13(3):jkac328. doi: 10.1093/g3journal/jkac328 (PMC10469399; doi:10.1093/g3journal/jkac328)
Supplement: jkac328_Supplementary_Data [file jkac328_supplementary_data.zip › Supplemental_Material_G3-2022-403852.docx]

**Supplementary**

**Genome assembly of *Thaumatotibia leucotreta*, a major polyphagous pest of agriculture in sub-Saharan Africa**

Anandi Bierman^1^*, Minette Karsten^2^ & John S. Terblanche^1^

*^1^Centre for Invasion Biology, Department of Conservation Ecology & Entomology, Stellenbosch University, South Africa; ^2^Department of Conservation Ecology & Entomology, Stellenbosch University, South Africa*

*corresponding author, e-mail: [anandie@sun.ac.za](mailto:anandi@sun.ac.za)


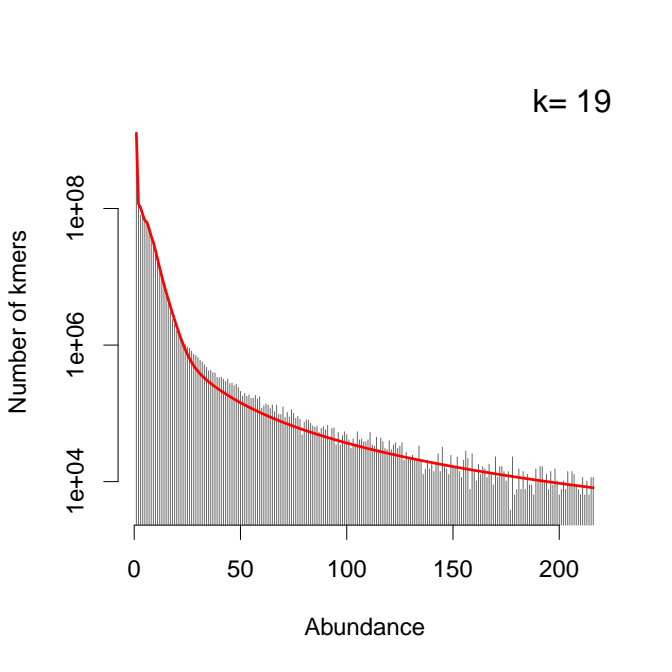


**Supplementary Figure 1** Kmer profile of best K of 19 for Illumina short reads as determined using Kmergenie v1.7016 (Chikhi and Medvedev, 2013).


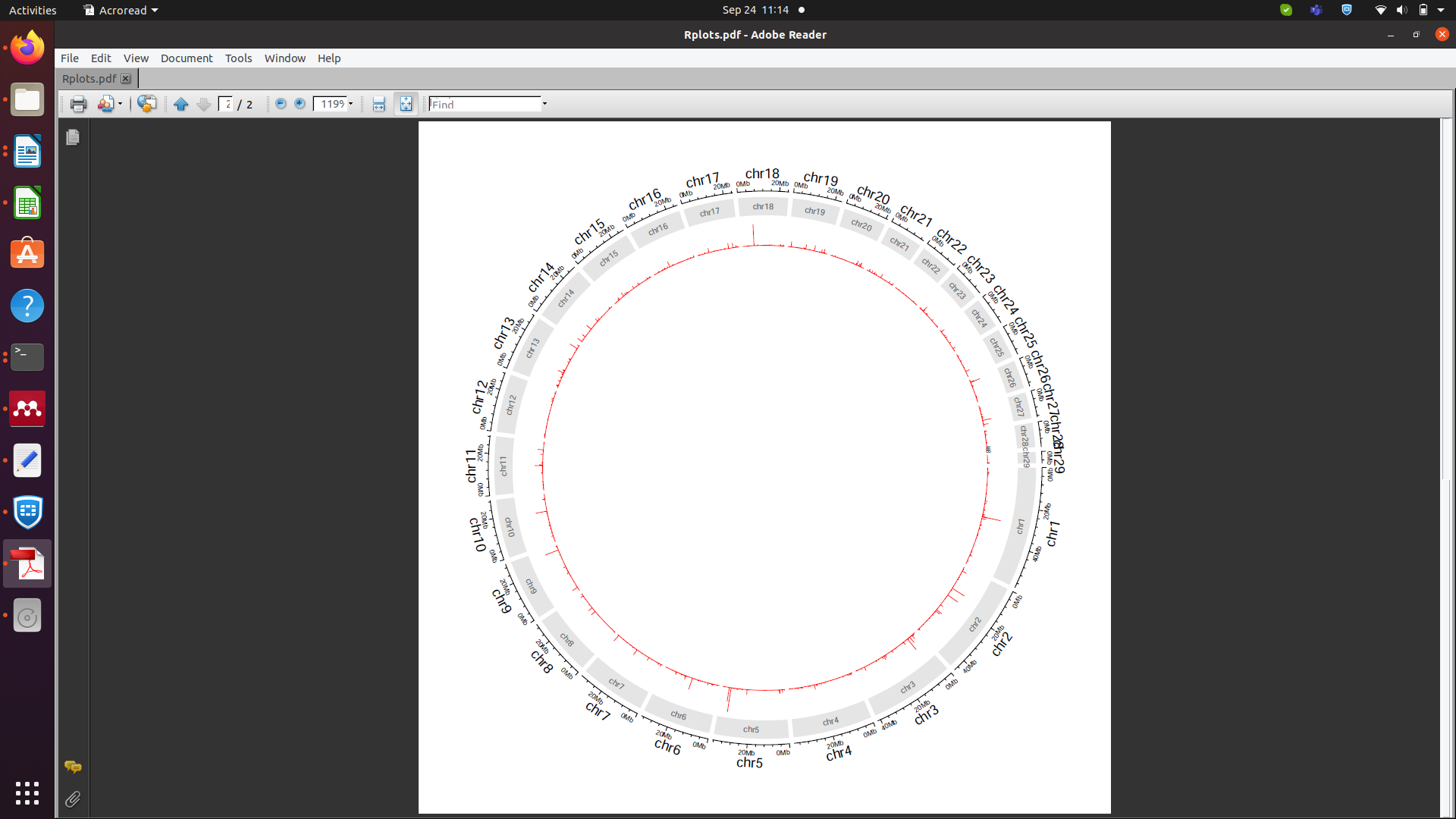


**Supplementary Figure 2** The chromosomal assembly of *C. pomonella* (outermost ring) mapped to the final FCM *T. leucotreta* assembly with coverage depicted in red (second ring). Peaks in the coverage are likely due to simple or repetitive genomic regions, sequenced at higher coverage. Graphic constructed using the *circlize* package in R.

**Supplementary Table 1**: Genome coverage calculated by dividing the number of bases sequenced (n) by the estimated genome size (g), multiplied by the percentage of reads placed in contigs (p).

| **Metric** | **Derived from:** | **Number:** |
| --- | --- | --- |
| Number of bases sequenced (n) | Bbmap count bases for PacBio reads and trimmed Illumina reads: | 9,013,598,515 |
| Estimated genome size (g) | QUAST assembly statistics: 804,903,742 | 804,903,742 |
| Percentage reads in contigs (p) | Canu assembler output:  Assembled sequences: 1440987  Total sequences:1607903 | 90% |
| Genome coverage | n/g*p | 10x |

**Supplementary Table 2**: Classes of repeat elements identified in the FCM assembly using RepeatMasker (v4.0.7).

| **Element category** | **Element sub category** | **Number of elements** | **Length occupied (bp)** | **Percentage of sequence (%)** |
| --- | --- | --- | --- | --- |
| SINEs |  | 64811 | 8749793 | 0,68 |
|  | ALUs | 27 | 1747 | 0 |
|  | MIRs | 90 | 4549 | 0 |
| LINEs |  | 407356 | 90133632 | 7,02 |
|  | LINE1 | 1871 | 119837 | 0,01 |
|  | LINE2 | 49295 | 13020837 | 1,01 |
|  | L3/CR1 | 16452 | 4776971 | 0,37 |
| LTR elements |  | 46612 | 20904939 | 1,63 |
|  | ERVL | 102 | 7900 | 0 |
|  | ERVL-MaLRs | 5 | 303 | 0 |
|  | ERV-classI | 1041 | 58151 | 0 |
|  | ERV-classII | 807 | 43778 | 0 |
| DNA elements |  | 142629 | 19170093 | 1,49 |
|  | hAT-Charlie | 39370 | 2389673 | 0,19 |
|  | TcMar-Tigger | 517 | 34517 | 0 |
| Unclassified |  | 86337 | 9117120 | 0,71 |
| Total interspersed repeats |  |  | 148075577 | 11,54 |
| Small RNA |  | 54560 | 6738623 | 0,53 |
| Satellites |  | 5018 | 532338 | 0,04 |
| Simple repeats |  | 316466 | 13838712 | 1,08 |
| Low complexity |  | 44555 | 2040280 | 0,16 |

**Supplementary Table 3**: Maker annotation pipeline (v2.31.10) (Cantarel et al. 2008) outputs. Protein homology evidence and EST evidence in the form of the peptide sequence file and CDS sequence file in fasta format of the *C. pomonella* genome assembly (Codling moth*;* Wan et al. 2019; GCA_003425675.2) was provided as input to the Maker pipeline.

| **Output** | **Total matches** | **Unique FCM contigs matched** | **Unique *C. pomonella* sequences matched** |
| --- | --- | --- | --- |
| Blastn: expressed_sequence_match | 7,428 | 3,179 | 7,428 |
| Blastx: protein_match | 1,252,212 | 13,254 | 15,871 |
| est2genome: expressed_sequence_match | 7,786 | 3,170 | 2,637 |
| CDS | 8,575 | 1,756 | NA |
| exon | 9,625 | 1,756 | NA |
| five_prime_UTR | 1,413 | 789 | NA |
| gene | 2,280 | 1,756 | NA |
| mRNA | 2,635 | 1,756 | NA |
| three_prime_UTR | 2,049 | 1,012 | NA |
| Protein2genome: protein_match | 1,095,854 | 12,865 | 14,395 |
