# Supplementary figures and images for: Genome assembly of Thaumatotibia leucotreta, a major polyphagous pest of agriculture in sub-Saharan Africa
Source: G3 (Bethesda). 2022 Dec 13;13(3):jkac328. doi: 10.1093/g3journal/jkac328 (PMC10469399; doi:10.1093/g3journal/jkac328)

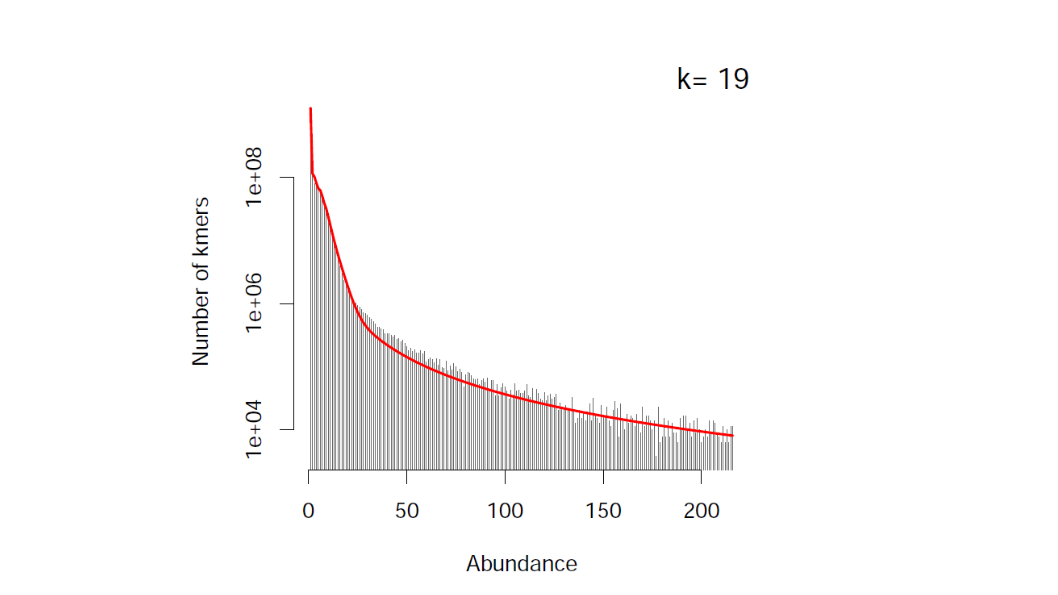

Supplement: jkac328_Supplementary_Data [file jkac328_supplementary_data.zip › Supplementary_Figure_1_G3-2022-403852.png]

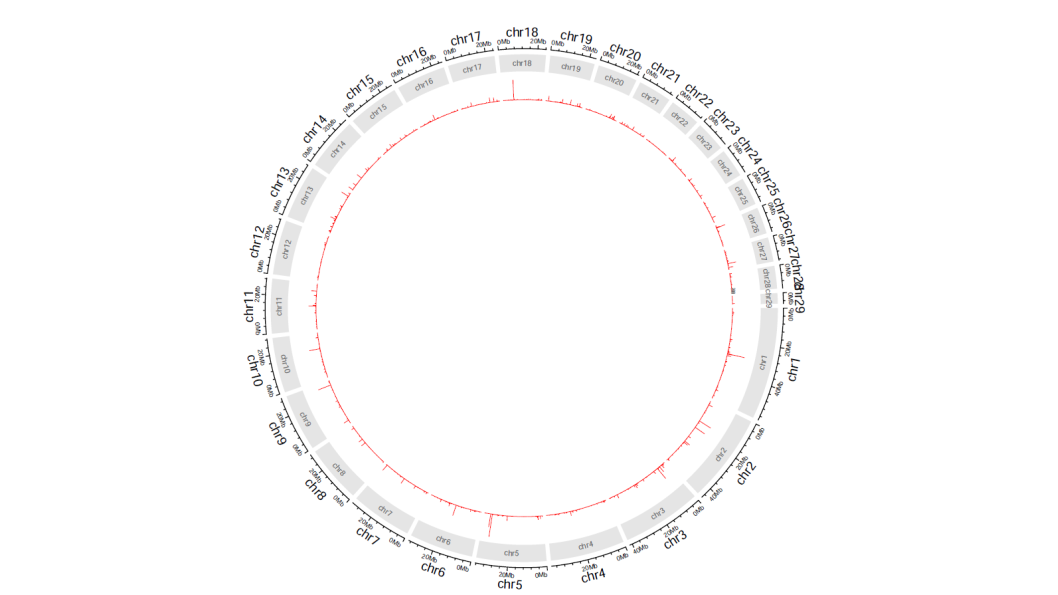

Supplement: jkac328_Supplementary_Data [file jkac328_supplementary_data.zip › Supplementary_Figure_2_G3-2022-403852.png]
